# Evolution of the highly networked deubiquitinating enzymes USP4, USP15, and USP11

**DOI:** 10.1186/s12862-015-0511-1

**Published:** 2015-10-26

**Authors:** Caitlyn Vlasschaert, Xuhua Xia, Josée Coulombe, Douglas A. Gray

**Affiliations:** Department of Biology, University of Ottawa, Ottawa, Canada; Department of Biochemistry, Microbiology and Immunology, University of Ottawa, Ottawa, Canada; The Ottawa Hospital Research Institute, Ottawa, Canada; Ottawa Institute of Systems Biology, Ottawa, Canada; Centre for Cancer Therapeutics, Ottawa Hospital Research Institute, 501 Smyth Road, Ottawa, ON K1H 8L6 Canada

**Keywords:** Ubiquitin-specific protease, Gene duplication, Gene phylogeny, Synteny, Subfunctionalization

## Abstract

**Background:**

USP4, USP15 and USP11 are paralogous deubiquitinating enzymes as evidenced by structural organization and sequence similarity. Based on known interactions and substrates it would appear that they have partially redundant roles in pathways vital to cell proliferation, development and innate immunity, and elevated expression of all three has been reported in various human malignancies. The nature and order of duplication events that gave rise to these extant genes has not been determined, nor has their functional redundancy been established experimentally at the organismal level.

**Methods:**

We have employed phylogenetic and syntenic reconstruction methods to determine the chronology of the duplication events that generated the three paralogs and have performed genetic crosses to evaluate redundancy in mice.

**Results:**

Our analyses indicate that USP4 and USP15 arose from whole genome duplication prior to the emergence of jawed vertebrates. Despite having lower sequence identity USP11 was generated later in vertebrate evolution by small-scale duplication of the USP4-encoding region. While USP11 was subsequently lost in many vertebrate species, all available genomes retain a functional copy of either USP4 or USP15, and through genetic crosses of mice with inactivating mutations we have confirmed that viability is contingent on a functional copy of USP4 or USP15. Loss of ubiquitin-exchange regulation, constitutive skipping of the seventh exon and neural-specific expression patterns are derived states of USP11. Post-translational modification sites differ between USP4, USP15 and USP11 throughout evolution.

**Conclusions:**

In isolation sequence alignments can generate erroneous USP gene phylogenies. Through a combination of methodologies the gene duplication events that gave rise to USP4, USP15, and USP11 have been established. Although it operates in the same molecular pathways as the other USPs, the rapid divergence of the more recently generated USP11 enzyme precludes its functional interchangeability with USP4 and USP15. Given their multiplicity of substrates the emergence (and in some cases subsequent loss) of these USP paralogs would be expected to alter the dynamics of the networks in which they are embedded.

**Electronic supplementary material:**

The online version of this article (doi:10.1186/s12862-015-0511-1) contains supplementary material, which is available to authorized users.

## Background

**Fig. 1:**
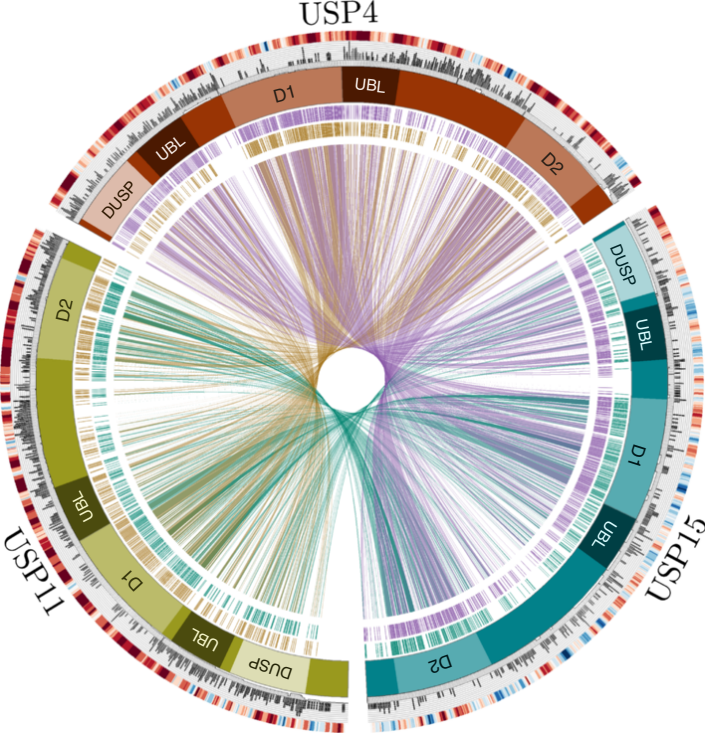
Comparison of USP4, USP15 and USP11 features. The red, blue and green boxes arranged in a circle represent USP4, USP15 and USP11, respectively. Domain structures are marked as follows: DUSP, domain in USP (N-terminal domain specific to these USPs); UBL, ubiquitin-like domain; D1 & D2, bi-part catalytic domain mediating ubiquitin cleavage. The interior of the circle links amino acid identities among paralogs, where each line represents an identical aligned residue. Links are colored as follows: USP4-USP15 purple; USP11-USP15 teal; USP4-USP11 gold. Alignment links are separated into two outer rings to facilitate viewing. The exterior of the circle features two rings illustrating the following: Inner ring: orthologous protein conservation. The histogram shows site-specific entropy among vertebrate species in black. High entropy reflects high dissimilarity. For comparative measure, the number of species containing the aligned region in question is below the histogram in gray. Low species count indicates amino acid indels. Outer ring: GC content. The heat map indicates relative GC content at the third codon position (GC3), where high GC content is red and low GC content is blue.

Protein ubiquitin tags are post-translational modifications that serve to either target substrates for proteasomal degradation or modify their interactive capacities [1]. Protein ubiquitination status is determined by the activities of the ubiquitin ligases that conjugate the ubiquitin moieties and the deubiquitinating enzymes (DUBs) that remove them; the balance of these activities thus affects key cellular processes. Among the most extensively networked [2] DUBs are the ubiquitin-specific protease (USP) paralogs USP4 and USP15, which regulate cell growth, embryonic development and innate immunity via their interactions with TGF-β [3, 4], Wnt/β-catenin [5] and NF-κB [6–8] pathway proteins respectively. USP4 and USP15 are also the only catalytic DUBs known to interact with the spliceosome [9–11], with more than eleven splicing factors identified as overlapping substrates [2]. This functional redundancy likely relates to their homology (there is 56.9 % amino acid identity in *Homo sapiens* as indicated in Fig. 1). One other DUB, USP11, bears considerable, albeit lesser, sequence identity to USP4 (44.5 % identity) and USP15 (43.2 %). The three paralogs share a common domain organization, consisting of a DUSP (*domain in USP*), two UBL (*ubiquitin-like*) and a bi-part catalytic domain (Fig. 1).

Overexpression of these DUBs has been noted in various human cancers, which may be attributable to their collective regulation of oncogenic proteins. For instance, all three paralogs regulate the type I TGF-β receptor while USP15 and USP11 also regulate several of its downstream effectors [4, 12, 13]. Conversely, whereas USP4 and USP15 target p53-inhibiting ligases ARF-BP1 [14] and MDM2 [15], respectively, USP11 stabilizes p53 [16] as well as several other tumor suppressors including PML [17], BRCA2 [18] and Mre11 complex members MRE11 & RAD50 [2]. In sum, though these paralogs are functionally redundant in some capacities, each appears to have undergone substantial subfunctionalization and neofunctionalization. A summary of their known protein interactions is presented in Table 2.

**Fig. 2:**
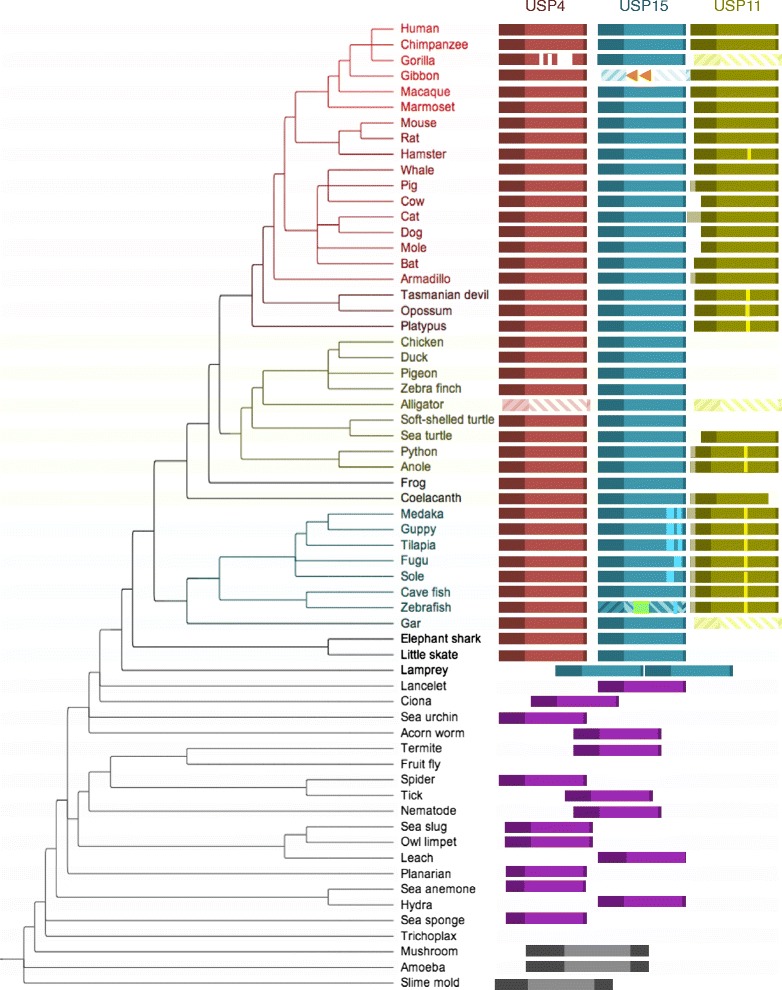
Phylogenetic distribution of USP4, USP15 and USP11. Red, blue, green, purple and black boxes represent USP4, USP15, USP11, ancestral (single copy) USP, and pre-USP ancestor sequences, respectively. Sequences are annotated and aligned according to their Reciprocal Best BLAST Hits (RBBHs), where lateral positioning of ancestral sequences indicates relative identity to human USP sequences. Translucent diagonally striped boxes indicate pseudogenes. Orange arrows indicate disruptive LINE1 element insertions in gibbon USP15 and green arrows indicate potentially disruptive insertion of a repetitive sequence of unknown origin in zebrafish USP15. Highlighted vertical bars indicate poly-glutamate sequences in USP15 and USP11

Functional versions of USP4, USP15 and USP11 are detectable in most branches of the vertebrate lineage including human. Of the three, USP4 and USP15 are most similar in terms of sequence identity (Fig. 1) and deubiquitination substrates (Table 2), which is consistent with the (USP11,(USP15,USP4)) branching pattern observed in phylogenetic analyses of these DUBs [19, 20]. This would suggest that the duplication that gave rise to USP4 and USP15 occurred most recently. However, a survey of USP paralogs encoded by metazoan genomes (Fig. 2) contradicts this hypothesis: while functional USP4 and USP15 are present in cartilaginous fish at the emergence of the gnathostome branch, USP11 is not identifiable until bony vertebrates make their appearance. What is more, all single-copy USP sequences have most identity with either USP4 or USP15. It is nevertheless possible that the USP11 duplication occurred earlier though its traces were erased by pseudogenization in deeper-branching species. One phylogeny represents the USP4, USP15 and USP11 relationship as a trifurcation [21], acknowledging its cryptic nature.

To understand the evolutionary changes in sequence, structure, and function among these paralogs, it is very important to know the temporal sequence of duplication. This enables us to determine which are the ancestral states and which are the derived states that potentially represent adaptation in response to an ancient environment. This motivated us to do phylogenetic studies to characterize the branching pattern and the timing of duplication events. An integrative *in silico* approach probing these systematic changes in a comparative genomic framework was employed to trace the duplication and subsequent radiation of USP4, USP15 and USP11. We first quantified and characterized USP paralogs in a set of representative metazoan genomes and delineated their divergence times in reference to known whole genome duplication events. We then evaluated ortholog variability to gain insight into the evolutionary processes that gave rise to the three paralogs observable in humans.

## Results

### Phylogenetics based on aligned nucleotide and amino acid sequence

**Fig. 3:**
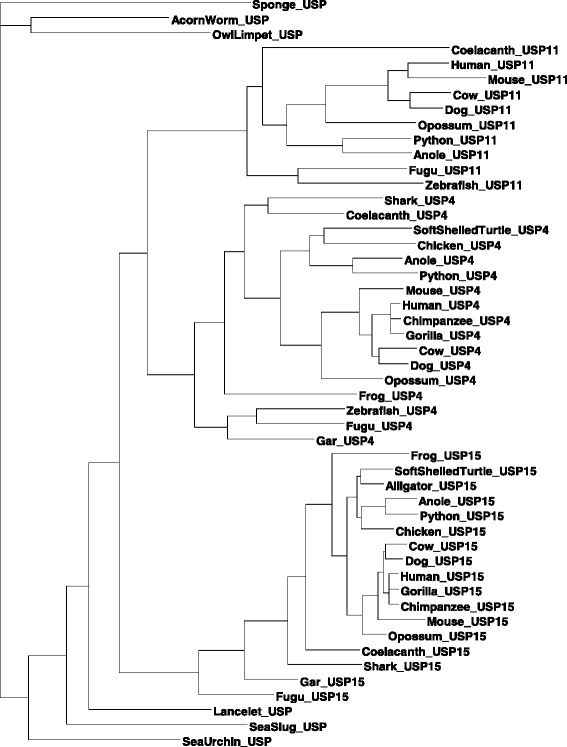
Maximum likelihood reconstruction of aligned codon sequences. A maximum likelihood tree of three paralogous genes from representative vertebrate species is represented together with their orthologs from invertebrate species. The unrooted tree was constructed with the F84 model and the maximum likelihood method implemented in DAMBE

**Fig. 4:**
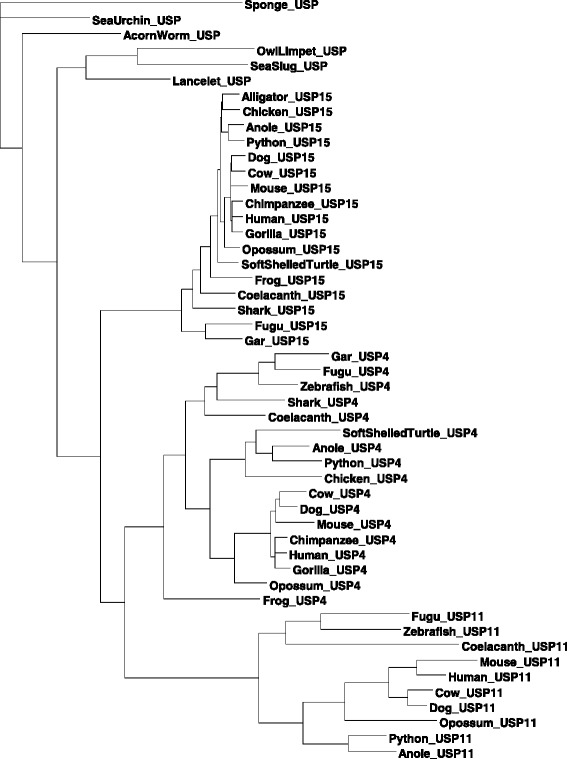
Maximum likelihood reconstruction of recoded amino acid sequences. Depicted is a maximum likelihood tree of 50 aligned amino sequences after recoding amino acids by size and polarity into four groups: small and polar (SCTND), large and polar (QEKRHY) small and non-polar (PAGV), large and non-polar (ILMFW). The rooted tree was produced using the ProtML method implemented in DAMBE

Fifty USP4, USP15 and USP11 coding sequences from 23 representative vertebrate and invertebrate genomes (listed in Table 1, Material and Methods) were aligned using MUSCLE [22] with Gblocks cleaning [23], yielding an aligned length of 3981 sites. For phylogenetic reconstruction, we used the maximum likelihood method implemented in DAMBE [24] with the estimated transition/transversion ratio, the F84 model, and *Amphimedon queenslandica* (sea sponge) as the outgroup. The resulting unrooted tree (Fig. 3) has drastically different evolutionary rates among different lineages, with USP11 evolving particularly faster than other lineages. We performed a likelihood ratio test of the molecular clock hypothesis with the 50 sequences and the TN93 model, and the clock hypothesis is strongly rejected (lnL without clock = −17452.3864, lnL with clock = −17630.0485, likelihood ratio chi-square = 355.3242, DF = 48, *p* < 0.0001). We have also tested the clock hypothesis by using the third codon positions only, but the clock hypothesis is still strongly rejected (lnL with no clock = −4053.1815, lnL with clock = −4116.3185, Likelihood ratio chi-square = 126.2739, DF = 48, *p* < 0.0001). Thus, the paralogous sequences are not appropriate for dating. Indeed, age-calibrated phylogenetic dating of the codon sequences generated a tree that placed the divergence of USP15 vertebrate sequences before that of the single-copy ancestral sequences (Additional file 1: Figure S1). This erroneous topology reflects a discord between the substitution model’s assumptions and the nature of the sequences: USP15 orthologs are situated in low-GC regions in vertebrates (human, mouse, chicken, lizard) while USP11 and USP4 are in moderately high-GC isochores. This bias is reflected in their respective GC3 contents (Fig. 1) and thus violates the fundamental assumption of time homogeneity of all practical substitution models. We note that the paralogous genes in vertebrate species are often located in different GC isochores [25, 26]. For this reason, a nucleotide-based or codon-based analysis may bias phylogenetic estimation. To address this problem, we have also analyzed aligned amino acid sequences of the 23 species by the likelihood method. We have adopted the approach recommended by Rodriguez-Ezpeleta et al. [27] by recoding amino acids by size and polarity into four groups: small and polar (SCTND), large and polar (QEKRHY), small and non-polar (PAGV), large and non-polar (ILMFW). This approach not only results in more robust phylogenetic reconstruction, but also dramatically reduces computation time. The resulting tree (Fig. 4) is largely concordant with the maximum likelihood tree topology based on aligned nucleotide sequences (Fig. 3), i.e., USP15 splitting first from USP4/USP11, followed by the USP4 and USP11 split, with the primitive species encompassing a single ortholog clustered close to the root.

**Table 1:** List of coding sequences analyzed with corresponding accession numbers

Species	USP4	USP15	USP11
Human	NC_000003	NC_000012	NC_000023
Gorilla	NC_018427	NC_018436	–
Chimpanzee	NC_006490	NC_006479	NC_006491
Rhesus monkey	NC_007859	NC_007868	NC_007878
Dog	NC_006602	NC_006592	NC_006621
Cat	NC_018724	NC_018729	NC_018741
Cow	AC_000179	AC_000162	AC_000187
Whale	NW_006725543	NW_006713252	NW_006727531
Mouse	NC_000075	NC_000076	NC_000086
Rat	NC_005107	NC_005106	NC_005120
Opossum	NC_008806	NC_008808	NC_008809
Little brown bat	NW_005872009	NW_005871371	NW_005871244
European shrew	NW_004545936	NW_004545859	NW_004545915
Star-nosed mole	NW_004567105	NW_004567135	NW_004567128
Armadillo	NW_004467831	NW_004502972	NW_004483933
Platypus	NW_001794469	NW_001688637	NW_001598857
Mallard	NW_004677124	NW_004676435	–
Zebra finch	NC_011476	NC_011463	–
Chicken	NC_006099	NC_006088	–
Alligator	–	NW_006225048	–
Python	NW_006532620	NW_006535771	NW_006532331
Green sea turtle	NW_006635848	NW_006644513	NW_006577128
Soft-shelled turtle	NW_005853649	NW_005858962	–
Anole	NC_014777	NC_014780	NC_014777
Xenopus	NW_004668239	NW_004668234	–
Coelacanth	NW_005819144	NW_005819645	NW_005821768
Gar	NC_023183	NC_023186	–
Zebrafish	NC_007117	NW_003336534	NC_007119
Zebra mbuna	NW_004531721	NW_004531746	NW_004531844
Guppy	NC_024335	NC_024353	NC_024337
Fugu	NC_018908	NC_018907	NC_018892
Medaka	XM_004068480	NW_004090515	NW_004095165
Shark	NW_006890068	NW_006890092	–
Lancelet	–	NW_003101526	–
Tunicate	–	NC_020166	–
Acorn worm	NW_003149765	NW_003123910	–
Sea urchin	NW_003577258	–	–
Sea slug	NW_004797520	–	–
Hydra	–	NW_004173592	–
Sea sponge	NW_003546314	–	–
Lamprey	lamprey_JL9400, lamprey_JL10812		
Little skate	LS-transcriptB2-ctg62960, LS-transcriptB2-ctg14739		
Owl limpet	gw1.79.7.1

The branching pattern of Figs. 3 and 4 enables us to infer an approximate time for gene duplication events. The USP15 lineage splits from (USP4,USP11) during the period between the divergence of vertebrates from primitive chordates (from 581 to 460.6 millions of years ago, or MYA [28]) and the branching of shark from teleost (462.5 to 421.75 MYA [28]), corresponding to the timing of a known whole genome duplication event [29]. A second gene duplication leads to the USP4/USP11 split which occurred in the common ancestor of bony fishes represented by gar, fugu, zebrafish and coelacanth (421.75 to 416 MYA [28]). USP11 is absent in shark. Given that the shark genome has evolved little [30], we may infer that the absence is ancestral instead of secondary loss, i.e., the USP4/USP11 split occurs after the divergence of shark from the ancestor of teleosts.

### Synteny of USP11 and USP4 loci supports duplication in a Euteleostome ancestor

**Fig. 5:**
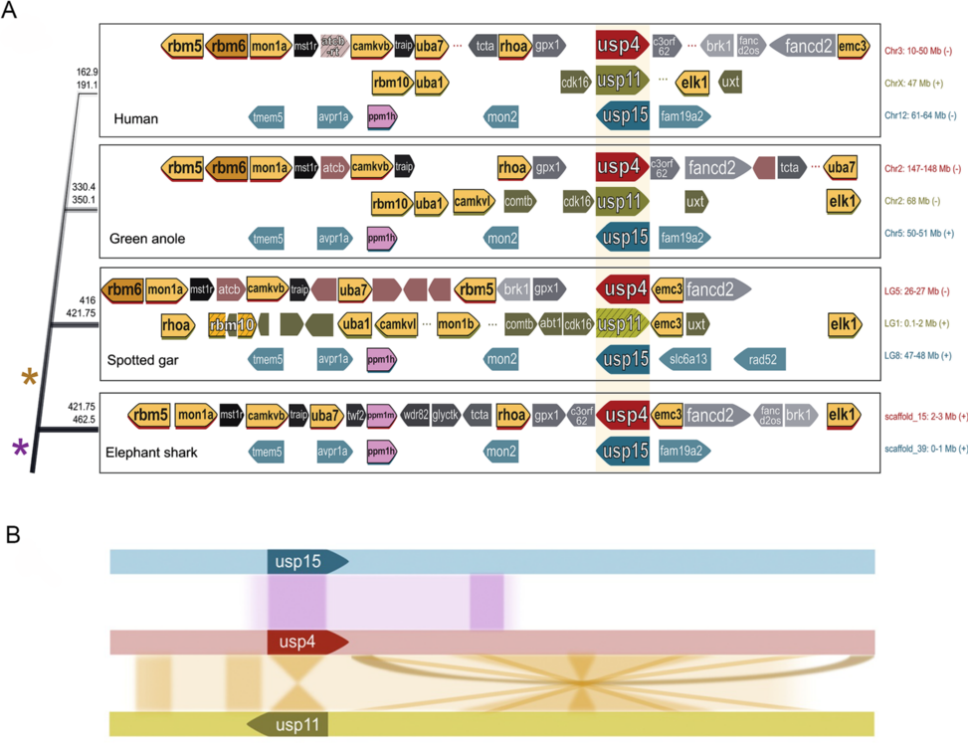
Shared synteny of USP4 and USP11 loci in Euteleosts. **a** Illustrated comparison of USP loci for elephant shark (*Callorhincus milii*), spotted gar (*Lepisosteus oculatus*), green anole (*Anolis carolinensis*) and human (*Homo sapiens*). Genes are represented by arrows, where black outlines indicate paralogous genes and striping indicates pseudogenes. Paralogs shared by USP4 and USP11 are coloured gold, while those shared by USP4 and USP15 are coloured purple. Genomic location of loci is indicated to the right. Upper and lower estimates of divergence times (in millions of years) indicated to the left for the following clades (in ascending order): jawed vertebrates (incl. shark), euteleosts (incl. gar), tetrapods (incl. anole) and mammals (human). Stars indicate inferred divergence times for USP4-15 (purple) and USP4-USP11 (gold). **b** Schema of paralogous gene collinearity and rearrangement events in (USP4-USP11) and (USP4-USP15) loci

Gene homologs often bear not only high sequence identity to their ancestors, but can also retain their genomic context. Synteny, the linear conservation of physically linked gene clusters within or between genomes, can be revelatory of paralogous or orthologous evolutionary relationships. We thus conducted a comparative analysis of the genomic region encompassing USP4 in *Callorhincus milii* (elephant shark) and the regions surrounding USP4 and the USP11 pseudogene in *Lepisosteus oculatus* (spotted gar), representing putative pre- and post-duplication loci. We found that several genes adjacent to shark USP4 map physically near to the USP4 orthologs in gar and other higher vertebrates including human and anole (Fig. 5). In fact, the synteny of the region is remarkably well conserved after duplication: in addition to USP11, six other functional paralogs of genes surrounding USP4 in shark and in gar can be identified within 1 Mb of gar pseudo-USP11, while these co-duplicates are absent from the shark genome. Invertebrate genomes likewise encode only a single copy of these genes. In contrast, no USP11 co-duplicates can be identified at the USP15 locus. This supports our inferred branching patterns (Figs. 3 and 4) and altogether suggests that a concerted duplication of the USP4 chromosomal region median to the divergences of gnathostomes and euteleostomes gave rise to USP11.

**Fig. 6:**
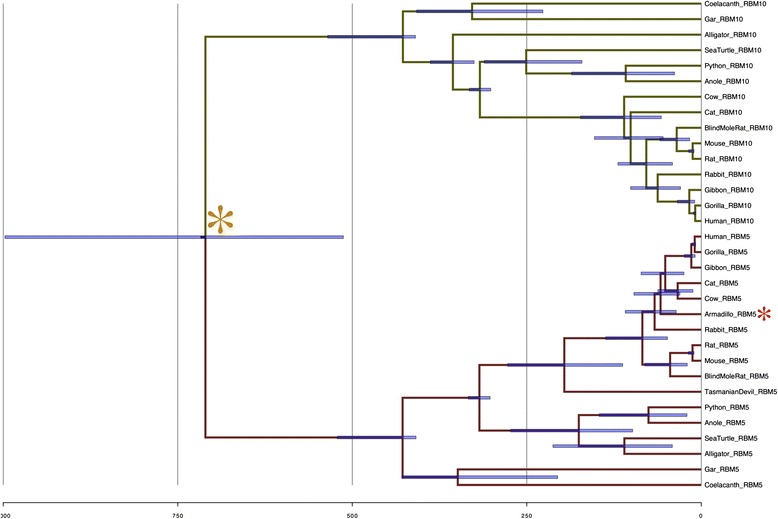
Bayesian dating of aligned co-duplicate codon sequences. Phylogenetic reconstruction and fossil-calibrated dating of aligned codon sequences for RBM5 and RBM10 was generated using BEAST v. 1.8. 95 % credible intervals are indicated. Calibration points were obtained from TimeTree. The gold star indicates the inferred RBM5-RBM10 divergence time. Red stars indicate major deviations from true topology

As a consequence of significantly different rates of evolution, Bayesian molecular dating of USP4 and USP11 aligned sequences overestimates their divergence time at 583–885 MYA. Three parallel runs of aligned USP4, USP11 and ancestral USP sequences with six calibration points converged at the rooted tree topology shown in Additional file 2: Figure S2 (note that several of the speciation node patterns and timing are largely inconsistent with known evolutionary relationships). Two sets of identified co-duplicates, RBM5/UBA7 and RBM10/UBA1, are co-localized with USP4 and USP11 respectively throughout vertebrate evolution, and can be used to date the duplication event by proxy. While neither RBM5/RBM10 nor UBA7/UBA1 follow a strict molecular clock, ∆lnL of RBM is greatly reduced compared to that of USP4 and USP11 (∆lnL_USP_ = 177.6621, ∆lnL_RBM_ = 77.8252; ∆lnL_UBA_ = 412.3984). Fig. 6 presents a phylogenetic reconstruction of RBM5 and RBM10 using a relaxed molecular clock; at 512 MYA, the 95 % credible interval upper bound of the predicted divergence time for these co-duplicates falls nearer the expected range and thus represents a rough estimate for the timing of duplication of the USP4 loci.

We believe our analyses provide overwhelming evidence in favor of a (USP15,(USP4,USP11)) branching pattern as opposed to the (USP11(USP4,USP15)) pattern that would be inferred based on sequenced relatedness [19, 20]. We posit that USP11 experienced greater coding sequence drift immediately following its duplication, resulting in complete pseudogenization in some species (e.g. gar) while in others a fast-evolving (Figs. 3 and 4), subfunctionalized (Table 2) protein emerged that is less similar than its well-conserved ancestors, USP4 and USP15. Adopting this novel understanding of their evolutionary relationship, we next examined the variability among USP homologs.

**Table 2:** Summary of USP4, USP15 and USP11 interaction partners

Pathway		USP4	Ref.	USP15	Ref.	USP11	Ref.
RNA splicing	Lsm2	+		+		−	
	Lsm4	+		+		−	
	Lsm6	+		+		−	
	Mepce	+		+		−	
	Naa38	+		+		−	
	Ppih	+		+		−	
	Prp3	+	[9]	+		−	
	Prp31	+		+		−	
	Prp4	+		+		−	
	Sart3	+	[9]	+	[10]	−	
	Tut1	+		+		−	
TGF-β signalling	Tgfbr1	+	[13]	+	[4]	+	[12]
	*Smad7*	−		+	[4]	+	[12]
	Smad1	−		+	[4]	−	
	Smad2	−		+	[4]	−	
	Smad3	−		+	[4]	−	
	Smad4	−		+	[4]	−	
	*Smurf1*	−		+		−	
	*Smurf2*	−		+	[4]	−	
	Bmpr1a	−		+	[81]	−	
Tumor suppression	p53	−		−		+	[16]
	*Mdm2*	−		+	[15]	−	
	*Arf-bp1*	+	[14]	−		−	
	Brca2	−		−		+	[18]
	Pml	−		−		+	[17]
	*Notch1*	−		+		+	
	Rb	+	[82]	−		−	
Innate immunity	Keap1	−		+	[83]	+	
	Rig-i	+	[84]	−		−	
	Trim25	−		+	[85]	−	
	Trim21	+	[86]	−		−	
	Rip1	+	[87]	−		−	
	Tak1	+	[6]	−		−	
	Traf6	+	[7]	−		−	
	Traf2	+	[7]	−		−	
	Ikka	−		−		+	[88]
	Ikba	−		+	[8]	+	[89]
Wnt/β-catenin signalling	Apc	−		+	[90]	−	
	Nlk	+	[5]	−		−	
Other	A2ar	+	[91]	−		−	
	Usp7	−		+		+	[92]

Interactors were identified in a mass proteomic analysis by Sowa et al. [2] and those validated by independent, small-scale studies are referenced. Note that these include K48- and K63-linked substrates and possibly non-substrate binding partners. Italicized interactors indicate proteins that have antagonistic roles in the indicated pathway

### Signature features of USP paralogs

**Fig. 7:**
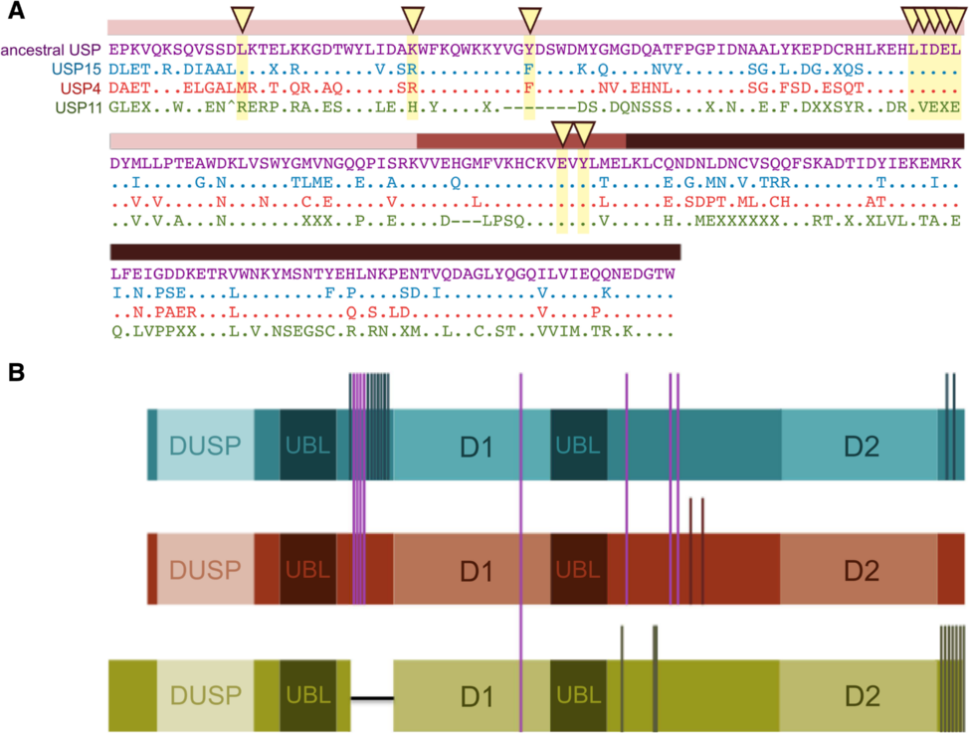
Summary of signature features of USP4, USP15 and USP1. **a** Alignment of signature sequences for USP4 (red), USP15 (blue) and USP11 (green) with the single-copy sequence (purple). The DUSP-UBL compound domain is shown and coloured as in Fig. 1. **b** Schematic illustration of signature phosphorylation sites and loss of alternatively spliced exon in USP11. Vertical bars traverse sequences with shared phosphorylation sites

Four key molecular traits are thought to engender paralog functional radiation: structure-function innovations, distinctive spliced isoforms, altered cellular regulation (via post-translational modification), and specific spatiotemporal expression patterns [31]. While the defining domain architecture presented in Fig. 1 is pervasive in all USP4, USP11 and USP15 as well as ancestral (single copy) homologs, divergence among the structured domains and the unstructured linking regions is observed, which has been reported to confer differential enzymatic properties [20, 32]. We herein derive the constitutive evolutionary differences, or molecular signatures, that have defined USP4, USP15 and USP11 from their inception using branching pattern knowledge and ancestral state reconstruction. Filtering paralog-defining features is more informative than monospecies sequence alignment, which contains intraparalog (species-specific) variations likely to be especially pronounced in the fast-evolving USP11. These conserved molecular signatures, explained below, are summarized in Fig. 7.

### Structure-function innovations

First let us consider the molecular signatures of structured regions in USP4, USP15, and USP11. The “domain in USP” (DUSP) and “ubiquitin-like” (UBL) structured regions form the N-terminal domain that distinguishes this subgroup of USPs. DUSP-UBL domains mediate some enzyme-substrate interactions [9, 33–35] and confer intrinsic regulatory capacities that have been structurally modeled for mammalian USP4, USP15 and USP11 [20, 32, 35, 36]. For instance, USP4 dimerization occurs in equilibrium through this domain, while neither USP15 nor USP11 are expected to dimerize in vivo [20]. The DUSP-UBL domain of USP4 also regulates ubiquitin active- site binding dynamics through its association with the unstructured insert region [32], though the absence of key residues impedes this regulatory function in human USP11 [32, 35]. The enzyme kinetics of USP15 are more similar to that of USP4 [32]. Given our derivation of their duplication chronology, it seems likely that the loss of ubiquitin-exchange regulation in USP11 is a derived and not ancestral state, though structural information is available only for mammalian proteins. Fig. 7a presents an alignment of the DUSP-UBL domains of an ancestral USP with the signature sequences of USP4, USP15 and USP11. Lancelet was selected as the ancestral species because it is the closest single-copy relative (Fig. 2). In addition, the domain sequence is identical in *Branchiostoma floridae* and the newly sequenced *B. belcheri*, two lancelets that have experienced a high degree of protein evolution [37], suggesting that it is an accurate depiction of a pre-duplication USP. USP signature sequences indicate residues that are conserved in a majority of members from each phylogenetic clade (elimination of species-specific substitutions). While the key residues are largely conserved in USP4, USP15, and the ancestral USP, disruption of the hydrophobic pocket and shortening of DUSP-UBL linker [32, 35] are signatures of USP11. This derived state implies that USP11 has had a different mode of action throughout time.

The two parts of the structured catalytic domain of these USPs, D1 and D2, are the most highly conserved regions among paralogs and orthologs (Fig. 1). Both are required for catalytic activity, and their conservation extends beyond the USPs under current consideration to the entire USP subfamily of deubiquitinating enzymes.

### Distinctive spliced isoforms

Whereas the seventh exon (E_7_) is alternatively spliced in USP4 and USP15, a corresponding exon is absent in USP11. The flexible linker region separating the DUSP-UBL and catalytic domains is roughly 20 residues long in USP11, its length in USP4 and USP15 short isoforms and the minimal length required for the aforementioned domain interaction [32]. Shark USP4 and the lancelet single-copy USP, ancestral to USP11, contain E_7_; what is more, both long and short isoforms have been reported in chondrichthyes. Thus, the “permanent skipping” of E_7_ in all USP11 represents a derived state. Alterations in the stoichiometry of USP4 isoforms have been reported for a rare bone disease [38], though the functional consequences of E_7_ alternative splicing have not been studied. In all species, the polypeptides encoded by USP4 and USP15 E_7_ are serine-rich, and many serve as putative post-translational modification (PTM) sites as identified in large-scale studies on human proteins [39]. In sum, the loss of E_7_ is a signature derived state of USP11 with potential functional or regulatory implications.

### Altered cellular regulation

Post-translational modification (PTM) regulation can differ among gene duplicates. Some PTM sites are well conserved while others stably differentiate the USP paralogs in question. For one, Ser445 (a known Akt phosphorylation site [13]), there is conservation in all USP4, USP11, USP15 and ancestral homologs. There are, on the other hand, multiple cases wherein a putative phosphorylation site has been lost or gained in USP11 relative to its ancestor, USP4. Within the insert region, USP4 Ser675 and Ser680 (identified phosphorylation substrates in multiple studies [40–48]) are conserved in USP15 but absent from USP11. Similarly, in an alignment of all USPs, putative phosphorylation site USP4 Tyr539 [39] is conserved in USP4 and USP15 while it is substituted by Phe in USP11. Slightly downstream, USP11 has Tyr551 (a reported phosphorylation site [39]) and Tyr554 whereas His and Phe, respectively, are universally present in USP4 and USP15. Still within the insert region, at positions 607 and 608, there exists in USP11 a pair of tyrosines that have been identified as phosphorylation sites in several large-scale studies [39]. The region in question aligns poorly with other paralogs, though there are two reported, albeit low confidence, serine phosphorylation sites in USP4 and none in USP15. As previously mentioned, the alternatively spliced exon, lost in USP11, contains several reported phosphorylated serines in USP4 and USP15 [39].

The N and C-termini are remarkably different among USP4, USP11 and USP15. The N-terminus of USP11 is longer, more disordered and more hydrophobic (rich in alanine). In addition, the C-termini of all gnathostome USP15 present a segment rich in aspartic acid, glutamatic acid and asparagine (e.g. human: 962-DEDSNDNDNDIENEN-976; shark: 978-DEDCNENDVENEN-990), except those of teleost fish, which instead have C-terminal segment(s) exceptionally rich in glutamate (e.g. zebrafish: 775-EKEEEEEDEDEEDVNDSEQEED-795; tongue sole: 966-DEEDEEEEEEEEGEVEEEDEEEEEGRE-981, 1015-NEREDEEEEEEEEEEEEEEEQE-1035). A poly-E repetitive sequence is also found in USP11 of various organisms, including teleost fish, some reptiles, the opossum and the Chinese hamster. These regions are schematically highlighted in Fig. 2. Aspartic acid and asparagine residues can be hydroxylated [49], though it remains to be seen whether any hydroxylation of such residues occurs within the acidic domains of the USPs. In addition, the D- & N- rich C-terminus of non-teleost fish USP15 presents two validated serine phosphorylation sites [39, 50–55], absent from USP4, whereas human USP11 has seven of these sites [39, 50, 55–57] within its final 20 residues that are conserved among mammals. While many of these conserved and differential phosphorylation sites remain to be functionally characterized, most are all located within unstructured regions, namely the insert, linker, and C-terminal regions. This is consistent with reports that disordered region often serve as PTM substrates [58–61] and changes in PTM regulation contributes to the functional divergence of paralogs [31]. In addition to phosphorylation and hydroxylation the disordered regions of the USPs may be subject to a number of other modifications including acetylation, methylation, and/or the addition of peptide moieties such as ubiquitin or SUMO. The contribution of this growing repertoire of PTMs to USP4, USP15, and USP11 regulation has yet to be established.

In sum, each paralog has distinctive signature features that represent common evolutionary categories namely structure-function innovations, distinctive spliced isoforms and altered cellular regulation. The fourth common differentiating trait, different spatiotemporal expression patterns, will be discussed in a later section.

### Variable mechanisms of USP11 loss

**Fig. 8:**
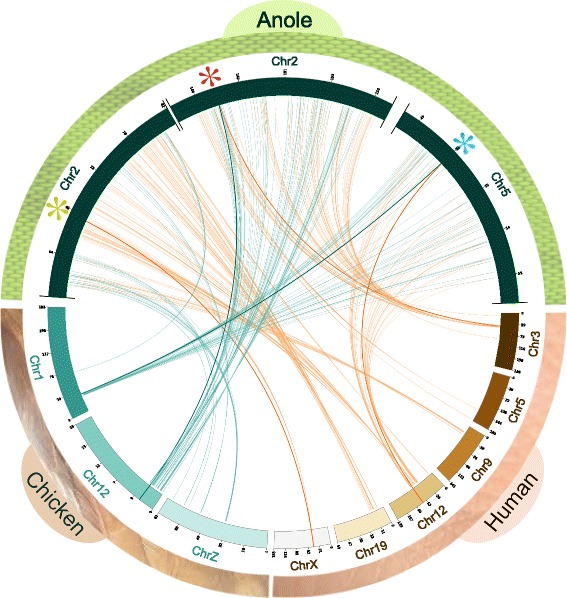
Synteny of USP4, USP15, and USP11 in anole compared to chicken and human. USP11 and surrounding region is absent in chicken. Asterisks represent the positions of anole USP genes (green indicates USP11, red is USP4, and teal is USP15)

As depicted in Fig. 2, USP11 has been lost multiple times throughout vertebrate evolution. In select fish, reptile and mammalian genomes, the syntenic loci where USP11 habitually resides hosts USP11 pseudogenes in lieu of functional genes. For instance, among reference primate genomes, USP11 is uniquely pseudogenized in *Gorilla gorilla*. The phylogenetic dispersal of pseudogenization suggests that these events occurred independently. In birds such as chicken, however, the entire syntenic region containing USP11 has been deleted while USP4, USP15 and their respective neighbouring genes are conserved, as illustrated in Fig. 8. A chromosomal rearrangement event in the avian ancestor may be responsible for the deletion of the segment containing USP11.

### In vivo demonstration of a minimal requirement for USP4 or USP15

Whereas the variable retention or loss of USP11 suggests that it is dispensable, all species contain either or both USP4 and USP15. It is reasonable to speculate that one functional copy from this gene pair is essential for viability, and we tested this hypothesis using mouse strains in which the *Usp4* or *Usp15* gene had been inactivated by the insertion of a retroviral provirus. The TF2497 and TF2834 strains have gene-trap proviruses in the *Usp4* and *Usp15* genes respectively; in both cases the insertion disrupts the gene near the 5′ end and precludes expression of a functional gene product (indeed no transcript can be detected by the sensitive method of reverse-transcription/polymerase chain reaction). Both strains are viable when homozygous for the inactivating insertion, and we have found no evidence of reduced fertility or obvious phenotypic effects. The lack of phenotypic consequences could be explained by functional redundancy between the USP4 and USP15 enzymes, but to determine if this is the case we conducted genetic crosses between mice heterozygous for the two genes (approval for these experiments was provided by the Animal Care Committee, University of Ottawa). As reported in Table 3, of 166 pups born we were unable to identify any progeny that had inactivating mutations in all four alleles, though all other expected genotypes were detected. Given that one of sixteen pups would be expected with the compound null genotype the lethality of this genotype can be asserted with a high level of confidence (from a binomial analysis *p* = (15/16)^166^ = 0.000022). We therefore conclude that USP4 and USP15 have sufficient functional redundancy to rescue inactivating mutations in a reciprocal fashion. The presence of functional USP11 genes is insufficient to rescue pups that are null for both USP4 and USP15. Mice are viable with one functional allele from the USP4/USP15 gene pair, though some apparent deviation from Mendelian ratios suggests that there may be phenotypic consequences of this haploinsufficient state. The nature of these consequences will be explored in future studies.

**Table 3:** Pooled progeny of USP4- and USP15-null mouse crosses

			USP 15		
		wt	het	null	*Total*
	wt	23	29	17	69
USP4	het	21	35	11	67
	null	8	22	0	30
	*Total*	52	86	28	166

Mice heterozygous for inactivating mutations of both USP4 and USP15 were mated, and progeny genotyped by polymerase chain reaction analysis. The number of progeny of each genotype is indicated, where wt represents homozygous wild-type, het represents heterozygous, and null represents homozygous null mice. The absence of progeny null for both USP4 and USP15 is statistically significant (*p* = 0.000022)

## Discussion

In the present work we have established the duplication chronology of a subgroup of highly networked ubiquitin-specific proteases, USP4, USP15 and USP11, and have characterized their subsequent radiation. According to the widely accepted 2R theory [29], vertebrate genomes have undergone two rounds of whole genome duplication (WGD). While it was conventionally assumed that these WGD events predated the divergence of jawless and jawed vertebrates [62], recent analysis of the elephant shark genome [30] placed at least one WGD event median to cyclostome and gnathostome divergence. In fact, subsequent studies have suggested that the 2R events occurred independently in cyclostomes and gnathostomes [63, 64], and that the former expansion was further shaped by an additional, lamprey-specific WGD. Thus, the USP15-like duplicate in lampreys is likely not orthologous to gnathostome USP4; rather, USP4 and USP15 appear to be ohnologs derived from WGD in a jawed vertebrate ancestor. In addition, the USP15-likeness of the lamprey version suggests that this paralog is the ancestral sequence, though there is no consensus among invertebrates as to whether their single copy most resembles USP4 or USP15 (Fig. 2). The issue cannot be settled by genomic synteny reconstruction, which is considerably more difficult in earlier species due to increased divergence time and the present lack of chromosomal assembly data for many species. In contrast, well-conserved intragenomic synteny points to the emergence of USP11 as the result of a more recent duplication event that does not coincide with any reported WGD event; it is likely the product of a small-scale duplication (SSD). The characterization of USP4/15 and USP4/11 duplications as WGD and SSD, respectively, corroborates well with reported trends for these phenomena: SSD-derived paralog sequences tend to evolve faster and are more functionally divergent [65].

Several notable differences in USP composition exist between and within clades. Primate species appear to have inconsistent USP repertoires: USP15 was inactivated by insertion of a LINE1 element in the gibbon, while erasure of USP11 and reduction of unstructured USP4 domains can both be observed in the gorilla. USP11 was also lost in the avian ancestor, inferred by the consistent absence of its genomic locus in all bird genomes (Fig. 8). Curiously, avian USP4 presents notable deviations from the signatures of this paralog: of the six bird genomes surveyed, all bear mutations in crucial residues for the ubiquitin-exchange mechanism [32], i.e. Arg40 and/or Met24 mutated in all, disruption of DUSP-UBL linker residues (a.a. 88–92) in chicken, QQD box region deleted in duck, and so on. In fact, USP4 is also more divergent in other species where USP11 was lost, which lead for example to the consistently incorrect branching patterns for frog and gorilla USP4 (Figs. 3 and 4; Additional file 1: Figure S1 and Additional file 2: Figure S2). What is more, avian USP4 adopts some USP11 signatures: these have collectively lost the Ser675 and Ser680 phosphorylation sites, while USP4 of the pigeon and zebra finch have also lost E_7_. The loss of these features that define all other USP4 (and USP15) is a derived state of USP11 and may thus represent a homoplastic convergence of avian USP4 toward the USP11 sequence.

Most of the signature features distinguish USP11 from USP4 and USP15, though the divergence of these last two is of practical interest due to their high protein sequence identity (Fig. 1), functional overlap (Table 2), and capacity for reciprocal rescue at the organismal level (Table 3). USP4 and USP15 differ in their codon usage: USP15, located in GC-poor isochores, employs more AT-ending codons than USP4. Low GC content is common in germ-line specific genes [25]. USP15 is in fact expressed at notably elevated levels in mature oocytes [66] (oocytes being the cell type for which its expression is the highest in mice [67]) while USP4 is at low abundance throughout oocyte maturation [68]. USP4 is predominantly expressed in somatic cells, particularly those of the immune system [67]. The distinct spatiotemporal expression patterns of USP4 and USP15 could explain why these redundant proteins have been maintained: vertebrate genomes could optimally encode two versions of an ancestral protein to accommodate its important roles in germ and somatic cells. While we show that one functional copy of USP4 or USP15 is a minimum requirement for viability (Table 3), the observed departure from Mendelian ratios may arise from a functional deficiency in oocytes haploinsufficient for USP15. Planned experiments (including in vitro culture of early embryos) should be informative in this regard. The expansion of TGF-β pathway substrates in USP15 may reflect an enhanced role in the regulation of oocyte development [69–71], while USP4 may have become the USP of greater importance in innate immunity pathways, as reflected by an increased number of substrates (Table 2). Further, an inserted in-frame zebrafish-specific repetitive element has modified the USP15 catalytic domain coding sequence of this species. While it remains to be seen whether the enzymatic activity of USP15 has been altered or inactivated by this insertion, we anticipate that perturbation of USP15 will provide insights into DUB network rewiring in the zebrafish. As a model system that is amenable to the testing of hypotheses through genome manipulation, the zebrafish should be ideal for future investigations of the respective roles and expression patterns of USP4 and USP15. The expression pattern of USP11 is notably distinct: without exception in human, mouse, rat, and pig its expression is predominantly neuronal [67]. In contrast to its paralogs [4], USP11 exerts a protective effect in glioma [17] as it stabilizes many tumor suppressors (Table 2).

**Fig. 9:**
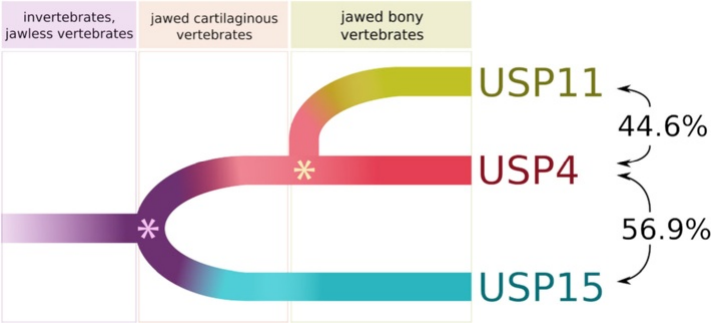
Phylogeny of USP genes. Percentages refer to amino acid identity between indicated USPs after global alignment. The pink asterisk denotes a whole genome duplication event, and the yellow asterisk denotes a small-scale duplication event involving USP4 and surrounding genes

While all organisms minimally retain USP4 or USP15 and some have in addition USP11, none have more than these three closely related USPs (including teleost fish and lamprey, which have experienced a third whole genome duplication). Genomes coding for USP4, USP15 and USP11 may thus represent the optimal system, where USP11 is an optional descendant whose functional contributions remain largely unexplored. Prior to the advent of whole genome sequencing it would have been reasonable to predict that with increasing organismal complexity there would be increasing complexity in molecular systems essential for development and tissue homeostasis, and the machinery relating to ubiquitin conjugation and removal would be high on the list of molecular systems expected to become more elaborate. In the case of deubiquitinating enzymes such a prediction would have been validated by whole genome sequencing: whereas vertebrate genomes encode more than 50 USP enzymes, roughly half this number are encoded by the genome of the fruit fly, and roughly half again by the genome of the budding yeast. In the evolution of complex molecular pathways the additional USP genes generated by WGD or SSD events could have provided substrate material for neofunctionalization or subfunctionalization. One can easily imagine how innovation within an augmented USP repertoire could facilitate innovation in complex signaling cascades (as exemplified by the NF-κB pathway central to innate immunity, or the TGF-β pathway). While we have restricted our focus to USP4, USP15, and USP11 we believe our analysis of the evolutionary history of this subset of deubiquitinating enzymes has been instructive in a broader sense. It demonstrates, for example, that BLAST alignments, while intuitive, can be misleading in the construction of an accurate USP phylogeny. Sequence similarity alone would not predict the branching pattern summarized in Fig. 9, which arose from extensive phylogenetic reconstruction of the DUSP-containing USP family incorporating aligned nucleotide and amino acid sequences, taxonomic distribution and patterns of synteny. We are hopeful that our approach can serve as a template for future studies of USP gene evolution, and will ultimate lead to a better understanding of the origins of this important gene family.

## Methods

### Sequence retrieval

Identification and proper annotation of homologs in an array of species is a first essential step in studying the evolution of duplicated genes. Coding sequences were retrieved from GenBank [72] and from genome project databases [62, 73, 74] using the well-annotated human sequences for USP4, USP15 and USP11 as tBLASTn queries [75]. Reciprocal Best BLAST Hit (RBBH) annotation transfer was applied to unannotated genomes. Accession IDs for all sequences are below.

### Sequence entropy

The site-wise Shannon entropy of aligned vertebrate USP amino acid sequences was calculated using DAMBE [24]. The results were plotted as a histogram using Circos [76].

### GC content analysis

The seqinr package in R was employed to generate plots for the GC content of the third codon positions (GC3) of the *Homo sapiens* USP4, USP15 and USP11 coding sequences using a sliding window of width 10. A heatmap of GC3 content was generated using Circos [76].

### Species tree reconstruction

A taxonomic phylogeny was generated using PhyloT [77]. Paralog affiliations were attributed as per their RBBH (described in *Sequence retrieval*). Putative non-processed pseudogene loci were confirmed using GenScan [78]. The SynMap function in CoGe [79] enabled comparison of the synteny of USP neighbouring regions in *Anolis carolinensis, Homo sapiens,* and *Gallus gallus*, which was visualized using Circos.

### Phylogenetic analysis

USP4, USP15 and USP11 codon sequences were aligned using MUSCLE [22]. The maximum-likelihood method using estimated transition/transversion ratio and F84 model, as implemented in DAMBE [24], was used to derive a phylogeny rooted on *Amphimedon queenslandica*. Molecular clock analyses were also conducted using DAMBE [24].

### Divergence dating

Codon alignments were produced by the MUSCLE algorithm using default GBlocks parameters as implemented in TranslatorX [22]. AIC and LRT nucleotide substitution model tests in DAMBE [24] designated the Generalized Time Reversible (GTR) as most appropriate for all alignments. BEAST v.1.8.2 [80], a Bayesian Markov chain Monte Carlo (MCMC)-based phylogenetic dating program, was employed to quantify USP age-calibrated divergence times. All analyses used a log normal relaxed molecular clock. The USP4/USP11 analysis used six calibration points obtained from TimeTree [28] (in millions of years): Dog-Cow[USP4,USP11]: 60, Human-Opossum[USP4]: 112, Human-Anole [USP4, USP11]: 320 & Gar-Human [USP4]: 418. The RBM5/RBM10 analysis employed four pairs of calibration points: Human-Gorilla [RBM5,RBM10]: 8, Mouse-Rat[RBM5,RBM10]: 10.4, Human-Anole[RBM5,RBM10]: 320 & Human-Zebrafish [RBM5,RBM10]: 425. Tracer was used to verify similar convergence after 20 million steps for 3 runs in each case.

### Mouse genetic crosses

TF2497 and TF2834 strains were purchased from Taconic Laboratories (Germantown, New York, USA), and were housed in a barrier facility at the University of Ottawa under protocol ME-305, approved by the Animal Care Committee, University of Ottawa. The strains were crossed to obtain mice heterozygous for proviral insertions in both the *Usp4* and *Usp15* genes. Eight pairs of compound heterozygous mice were mated under standard conditions, and progeny were obtained for genotyping. Genotyping was performed at 3–4 weeks of age, using tissue from ear punches. DNA was prepared using the REDExtract-N-Amp™ Tissue PCR Kit (Sigma-Aldrich Canada, Oakville, Ontario) and polymerase chain reaction was performed using the kit reagents. For genotyping of USP4 the forward primer used was derived from the third exon (upstream of the proviral insertion site): 5′- CCAGCAGCCTATTGTCAGAA -3′, where reverse primers were derived from the third intron (downstream of the proviral insertion site): 5′- TCAGTACTTAGGGATCTCTGA -3′ or from the neomycin phosphotransferase gene within the provirus: 5′- AACCTGCGTGCAATCCATCT -3′. Amplification conditions for USP4 were as follows: initial denaturation at 95C for 3 min followed by 30 cycles of 95C for 30 s, 57C for 30 s and 72C for 60 s and a final cycle at 72C for 5 min. A PCR product of approximately 250 base pairs was generated from the wild type gene, whereas the disrupted gene generated a product of approximately 1000 bp as detected by ethidium bromide staining of 1 % agarose gels. For USP15 a similar strategy was adopted using the forward primer: 5′ – GGTTTGAAGGATAACGTAGGC -3′, and reverse primers 5′ – ATAAACCCTCTTGCAGTTGCATC -3′ and 5′- GAGTACCTAACAGGCACTTGAGACG -3′. USP15 PCR conditions were similar except that annealing was done at 55C for 30 s and elongation at 72C was reduced to 45 s.

## Availability of supporting data

All supporting data are included as additional files in the form of Additional file 1: Figure S1 and Additional file 2: Figure S2.

## Additional files

Additional file 1:
**Figure S1.** Bayesian dating of aligned codon sequences. Phylogenetic reconstruction and fossil-calibrated dating of aligned codon sequences for USP4, USP15, USP11 and ancestral USP sequences was generated using BEAST v. 1.8. 95 % credible intervals are indicated. Calibration points were obtained from TimeTree. (PDF 64 kb) Additional file 2:
**Figure S2.** Bayesian dating of aligned USP4 and USP11 codon sequences. Phylogenetic reconstruction and fossil-calibrated dating of aligned codon sequences for USP4 and USP11 was generated using BEAST v. 1.8. 95 % credible intervals are indicated. Calibration points were obtained from TimeTree. The gold star indicates the inferred USP4-USP11 divergence time. Red stars indicate major deviations from true topology. (PDF 52 kb)
