# Supplementary figures and images for: Evolution of the highly networked deubiquitinating enzymes USP4, USP15, and USP11
Source: BMC Evol Biol. 2015 Oct 26;15:230. doi: 10.1186/s12862-015-0511-1 (PMC4624187; doi:10.1186/s12862-015-0511-1)

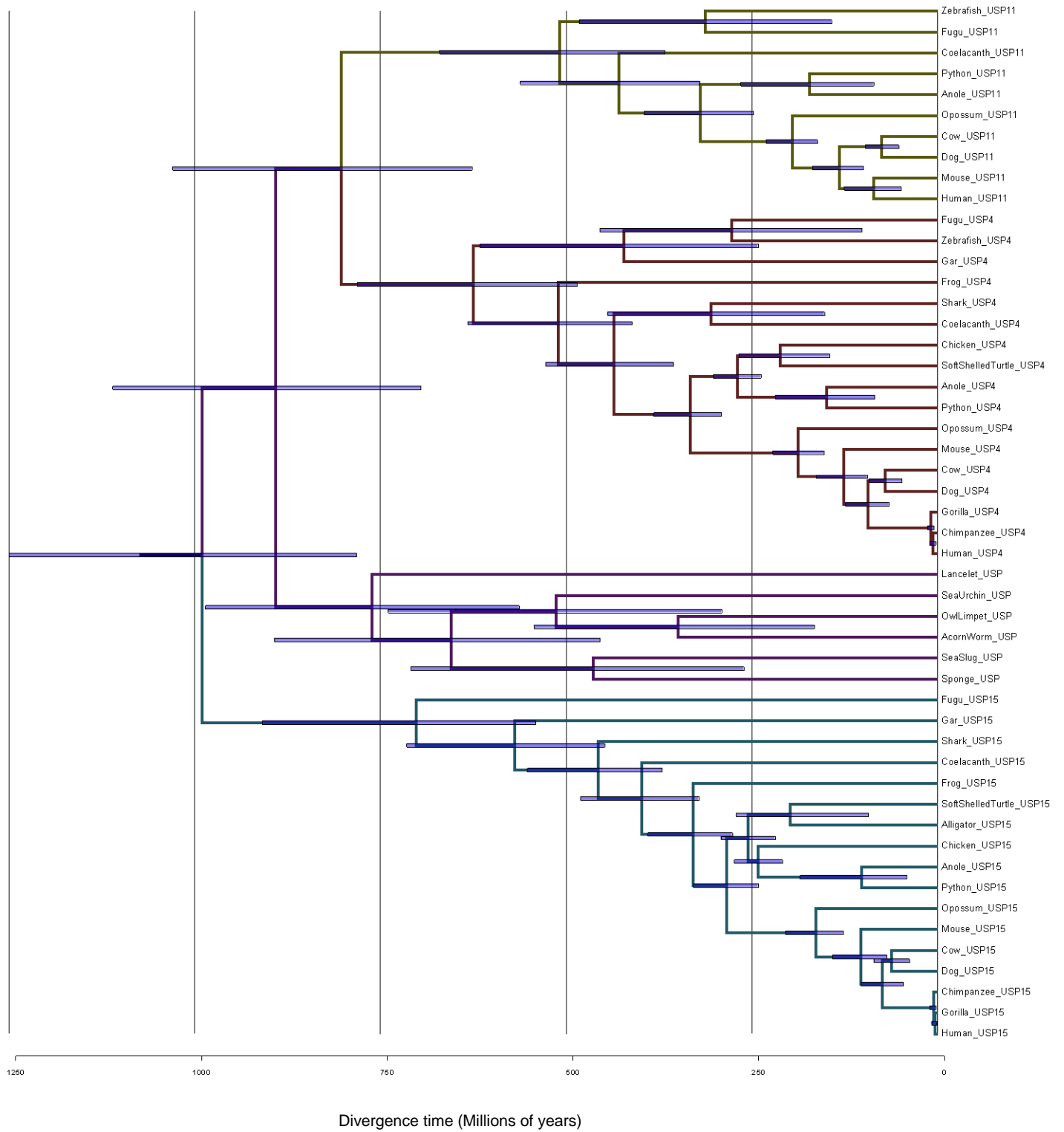

Supplementary Figure 1

Supplement: Additional file 1: — Figure S1. Bayesian dating of aligned codon sequences. Phylogenetic reconstruction and fossil-calibrated dating of aligned codon sequences for USP4, USP15, USP11 and ancestral USP sequences was generated using BEAST v. 1.8. 95 % credible intervals are indicated. Calibration points were obtained from TimeTree. (PDF 64 kb) [file 12862_2015_511_MOESM1_ESM.pdf]

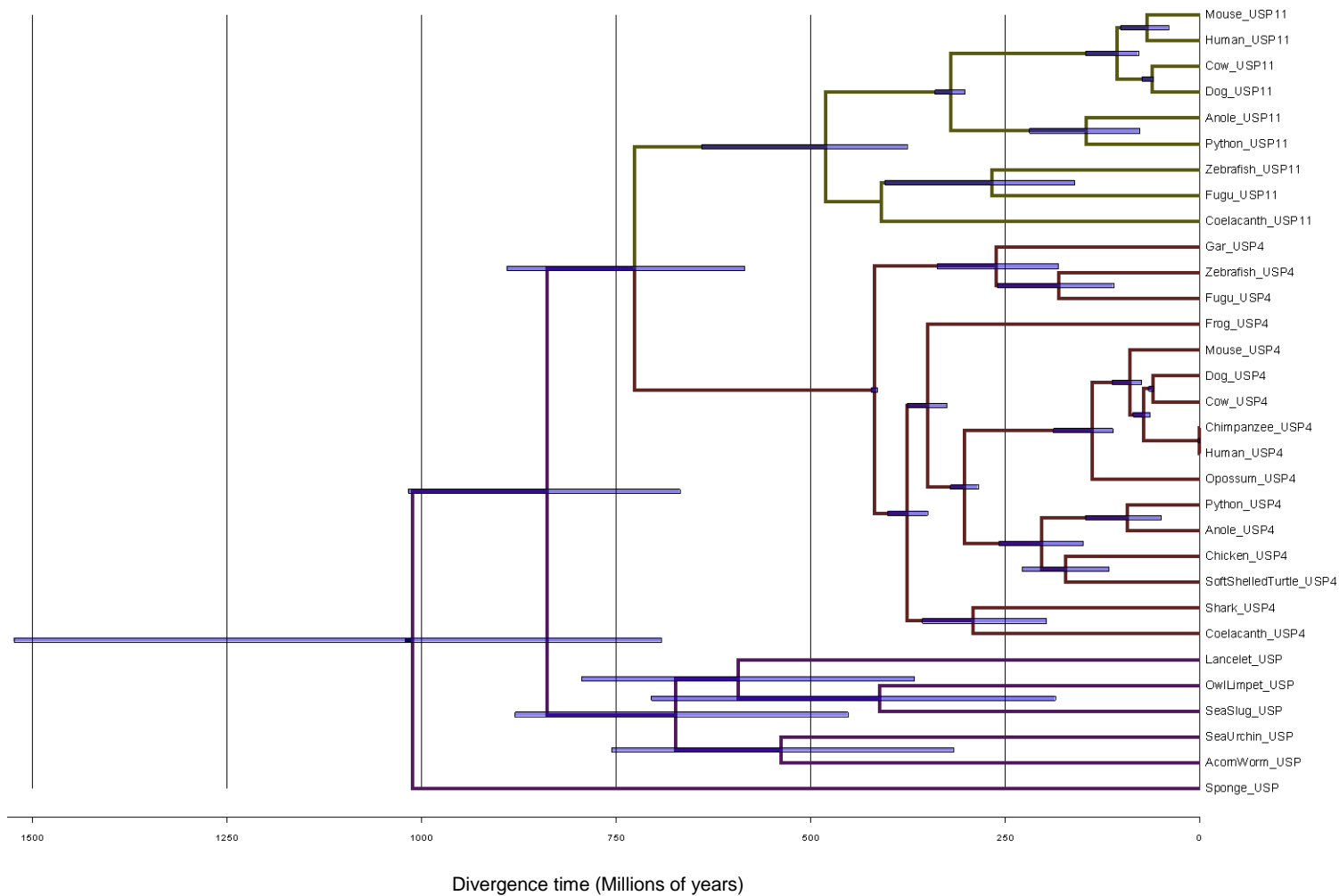

Supplementary Figure 2

Supplement: Additional file 2: — Figure S2. Bayesian dating of aligned USP4 and USP11 codon sequences. Phylogenetic reconstruction and fossil-calibrated dating of aligned codon sequences for USP4 and USP11 was generated using BEAST v. 1.8. 95 % credible intervals are indicated. Calibration points were obtained from TimeTree. The gold star indicates the inferred USP4-USP11 divergence time. Red stars indicate major deviations from true topology. (PDF 52 kb) [file 12862_2015_511_MOESM2_ESM.pdf]
